# Integrated Single-Cell RNA-Seq and Machine Learning to Construct an EMT Infiltration Scoring Model for Prostate Cancer

**DOI:** 10.3390/ijms27115017

**Published:** 2026-06-02

**Authors:** Zhipeng Xie, Yingjie Sun, Yuheng Tang, Qi Qi, Jiaxiang Liang, Jiehui Zhang, Wenru Tang, Xuhong Zhou

**Affiliations:** 1 Tumor and Aging Laboratory, Kunming University of Science and Technology, Kunming 650500, China; 13977391933@163.com (Z.X.);; 2 Office of Science and Technology, Yunnan University of Chinese Medicine, Kunming 650500, China

**Keywords:** biochemical recurrence, epithelial–mesenchymal transition, immune checkpoint, prostate cancer, tumor immune microenvironment, copy number variation (CNV), machine learning, single-cell analysis

## Abstract

Prostate cancer (PCa) remains a major global health concern, with a subset of patients progressing to aggressive disease despite advances in diagnosis and treatment. Epithelial–mesenchymal transition (EMT) plays a pivotal role in tumor invasion, metastasis, and immune evasion; however, its cellular heterogeneity and clinical relevance in PCa remain incompletely understood. We analyzed single-cell transcriptomic data to characterize EMT dynamics in malignant epithelial cells. Malignant cells were identified based on aberrant copy number variation patterns, and EMT activity was quantified using AUCell. Gene expression profiling and gene set enrichment analysis identified key EMT-associated genes. By integrating bulk transcriptomic data with LASSO regression analysis, we identified five pivotal genes and constructed an EMT infiltration scoring model. The model demonstrated robust predictive performance in an external Gene Expression Omnibus validation cohort and effectively predicted early biochemical recurrence. Further analyses revealed significant associations between EMT scores, clinicopathological features, immune cell infiltration, genomic instability, and tumor immune dysfunction and exclusion scores. Pathway enrichment analysis highlighted distinct molecular characteristics between high- and low-score groups. Additionally, molecular docking using AutoDock identified potential targeted therapeutic agents for key EMT genes. Overall, this study systematically delineates EMT heterogeneity at the single-cell level and establishes a robust EMT infiltration model for prognostic prediction and therapeutic guidance in PCa, providing novel insights for precision risk stratification and individualized treatment strategies.

## 1. Introduction

Although PCa is one of the most common malignancies among men, a major clinical challenge remains the substantial risk of post-treatment recurrence and progression to lethal disease [1]. Although advances in surgery, radiotherapy, androgen deprivation therapy, and novel endocrine and chemotherapy regimens have significantly improved the overall prognosis of patients with localized PCa during the last decade, a substantial proportion of patients still experience BCR or progress to castration-resistant prostate cancer (CRPC), both of which are associated with poor clinical outcomes [2]. This phenomenon suggests that traditional assessment systems based on anatomical staging and pathological features are insufficient to fully capture the biological complexity of PCa. Elucidating the key molecular mechanisms underlying tumor progression is essential for improving patient risk stratification and guiding precision therapy.

EMT, a pivotal cellular program, facilitates tumor invasion, metastasis, and therapeutic resistance by reducing intercellular adhesion, enhancing cellular motility and invasiveness, and increasing adaptability to hostile microenvironments [3]. Emerging studies suggest that EMT critically contributes to the malignant progression of a range of solid tumors [4]. In PCa, enhanced EMT-related molecular features are generally associated with increased invasiveness, adverse clinical outcomes, and potential therapy resistance, highlighting the functional importance of EMT in driving tumor biological behavior [5].

Functionally, EMT constitutes a critical cellular program involving diverse phenotypic and functional changes, rather than a single molecular event. Tumor cells with varying EMT activity levels may exhibit significant differences in invasive potential, pathway activation patterns, and interactions with the tumor microenvironment [6]. However, existing studies on EMT in PCa have largely relied on bulk transcriptomic data [7], which hampers the resolution of EMT activity at the single-cell level and constrains the identification of functional features linked to distinct EMT states. This limitation restricts the application of EMT for tumor stratification and mechanistic studies, highlighting the need for a high-resolution, systematic characterization of EMT functional states and their associated biological features in PCa.

The development of scRNA-seq technology provides a powerful tool to dissect intratumoral heterogeneity [8], making it possible to assess EMT functional states at the single-cell level. Leveraging this technology, the present study integrated multiple PCa scRNA-seq datasets and quantitatively evaluated EMT activity in malignant epithelial cells identified as tumor-derived. These cells were further classified into subpopulations representing distinct functional states, and their molecular features and potential biological differences were systematically analyzed. By combining multi-cohort transcriptomic data, this study also constructed and validated an EMT-related functional scoring system intended to explore the functional impact of EMT on PCa progression and to develop a systematic approach for accurate patient stratification and tailored clinical management.

## 2. Results

### 2.1. Characterization of Epithelial Cells and Derivation of Core EMT Gene Features from Single-Cell Data

**Figure 1:**
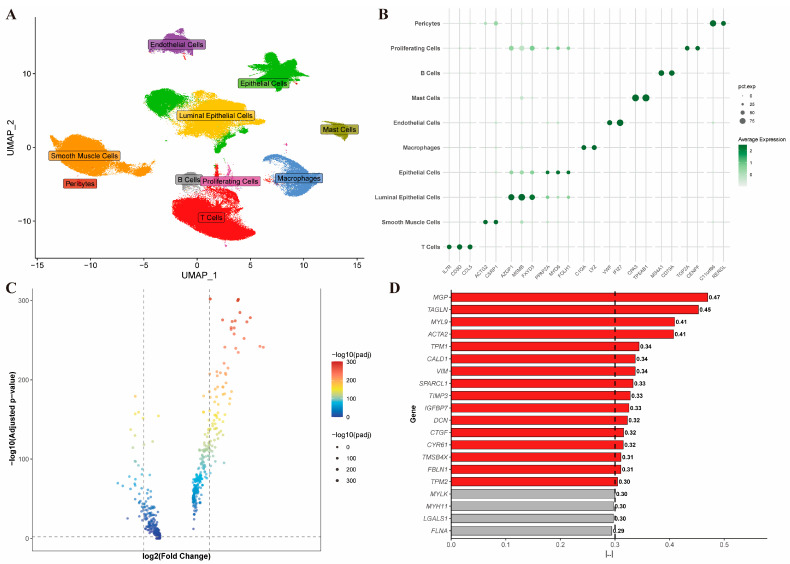
Identification of key EMT-related genes by single-cell transcriptome analysis. (**A**) UMAP dimensionality reduction showing cell clustering results and cell type annotation. (**B**) Expression distribution of representative marker genes in different cell subpopulations. (**C**) Volcano plot of DEGs in malignant epithelial cells between high- and low-EMT-score groups. (**D**) Bar plot of scores for key EMT-related genes identified by ssGSEA.

Following comprehensive preprocessing and quality assessment of the integrated single-cell transcriptomic data (Supplementary Figure S1), batch effect correction, dimensionality reduction, and unsupervised clustering, a total of 20 distinct cell clusters were identified [9]. Annotation of cellular identities was performed using well-recognized marker gene signatures, resulting in the classification of 10 major cell types, including epithelial cells, macrophages, T cells, and other immune and stromal populations (Figure 1A). The assigned cell populations showed specific expression signatures consistent with their corresponding marker genes, validating the reliability of the cell annotations (Figure 1B). To further characterize EMT-related transcriptional features in PCa epithelial cells, epithelial lineage-related cells, including epithelial and luminal epithelial cells, were extracted for downstream analyses.

Epithelial cells were further stratified into malignant and non-malignant subsets according to tumor-related transcriptomic features. CopyKAT was applied to the extracted epithelial lineage cells for CNV inference [10]. Based on genome-wide CNV profiles, epithelial cells were classified as malignant or normal epithelial cells. The CNV-based annotation was visualized using t-SNE, showing a relatively segregated distribution pattern of epithelial cells with different CNV states in low-dimensional space, reflecting transcriptional heterogeneity (Supplementary Figure S2A,B).

To quantify EMT-related transcriptional activity in malignant epithelial cells at the single-cell level, AUCell was used to score each cell based on EMT-related gene sets. An EMT activity score based on AUC values was used to classify cells into high- and low-EMT groups. Subsequent t-SNE analysis of malignant epithelial cells, annotated according to EMT activity, revealed partial overlap between EMT-high and EMT-low cells in low-dimensional space rather than complete separation (Supplementary Figure S2C,D). This partially overlapping distribution indicates heterogeneity in EMT-related functional states, providing a foundation for further functional characterization of EMT-associated biological differences [11].

Comparative transcriptomic profiling was performed between EMT-high and EMT-low malignant epithelial cells to identify differentially expressed genes. Statistical evaluation relied on a Wilcoxon-based nonparametric approach, followed by Benjamini–Hochberg correction to account for multiple comparisons and control false-discovery rates. Genes satisfying the criteria of adjusted *p*-value (*p*adj) < 0.01 and |avg_log2FC| > 0.5 were selected for subsequent functional analyses. Transcriptomic comparison revealed 516 genes with significant expression differences, with 289 genes upregulated in EMT-high cells and 228 genes upregulated in EMT-low cells (Figure 1C).

EMT-related pathway activity linked to the DEG set was quantified in individual cells using ssGSEA (v1.50.5) [12]. Based on ssGSEA scores, 16 core genes exhibiting significantly higher scores in EMT-high cells were identified as candidate genes for subsequent model construction (Figure 1D).

### 2.2. Establishment and Performance Evaluation of the EMT Infiltration Scoring Model

An EMT infiltration scoring model was constructed to quantify tumor EMT activity and infiltration potential in prostate cancer patients. Tumor progression status was defined based on pT stage: patients with pT2 were classified as organ-confined disease, whereas pT3 and pT4 patients were classified as locally advanced disease [13]. The input features were candidate genes significantly associated with EMT activity, and the model output was a continuous risk score for evaluating tumor EMT status and early-recurrence risk.

To construct a stable model with robust generalizability, multiple public transcriptomic datasets were integrated, including the CGA-PRAD cohort (n = 502) and GEO datasets GSE62116 (n = 235) and GSE220095 (n = 176). TCGA-PRAD and GSE62116 were corrected using the ComBat algorithm to minimize potential batch effects, with effectiveness confirmed by PCA (Supplementary Figure S3). A random 70% of samples was assigned to the training set, with the remaining 30% forming the testing set, mixing samples from both datasets to enhance robustness and generalizability. GSE220095 was used as an independent external validation cohort to evaluate cross-cohort predictive performance.

Using the training set, LASSO logistic regression was applied to the 16 EMT-related candidate genes for feature selection. After cross-validation, five core genes—*IGFBP7*, *FBLN1*, *MGP*, *TPM2*, and *TIMP3*—were selected for model construction. EMT infiltration scores were computed according to the method described below:

*Risk score* = 0.6866 × *IGFBP7_Exp_* + (−0.5764) × *FBLN1_Exp_* + 0.3932 × *MGP_Exp_* + (−0.2000) × *TPM2_Exp_* + (−0.0274) × *TIMP3_Exp_*

**Figure 2:**
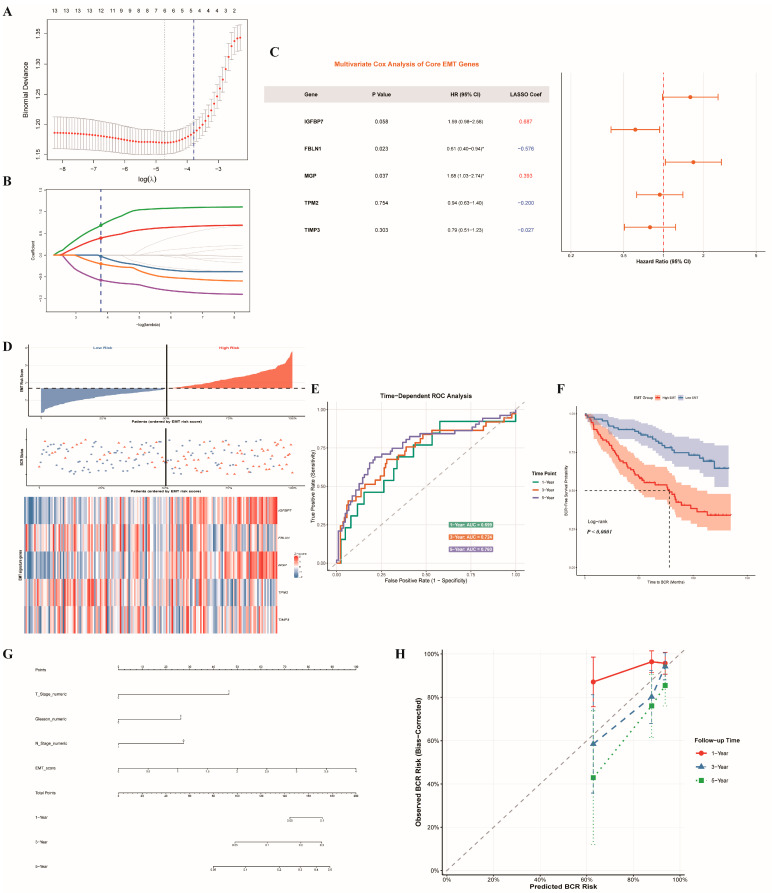
Construction and external validation of the EMT-related gene risk model. (**A**) LASSO regression cross-validation curve used to determine the optimal penalty parameter λ. (**B**) LASSO coefficient trajectory plot showing the shrinkage process of candidate gene coefficients as λ changes. (**C**) Multivariate Cox regression forest plot of model genes, displaying each gene’s independent prognostic effect and hazard ratio. (**D**) Distribution of patient risk scores, survival status, and heatmap of model gene expression in the external validation cohort, ordered from low- to high-risk scores. Circles indicate recurrence-free patients, whereas triangles indicate patients with BCR. (**E**) Time-dependent ROC curves for 1-, 3-, and 5-year survival in the external validation cohort. (**F**) Kaplan–Meier survival curves stratified by risk score. (**G**) Prognostic nomogram constructed based on risk score and clinical variables. (**H**) Calibration curves for the nomogram predicting 1-, 3-, and 5-year survival probabilities. * *p* < 0.05.

Multivariate Cox regression confirmed that each of the five genes contributed independently to the EMT infiltration score (Figure 2C), highlighting their key roles in model construction.

Model performance evaluation showed that the time-dependent receiver operating characteristic (ROC) area under the curve (AUC) was 0.762 in the training set and 0.776 in the testing set (Supplementary Figure S4A,B), indicating stable predictive ability. The AUC for the model in the external validation cohort was 0.696 (Supplementary Figure S4C), suggesting moderate predictive performance across different cohorts despite potential inter-cohort heterogeneity.

To further validate the clinical relevance of the EMT infiltration score, BCR was selected as the survival endpoint, reflecting early tumor recurrence and invasiveness [14]. In the GSE220095 cohort, patients were stratified into high-scoring and low-scoring subgroups based on their EMT infiltration scores. Distinct patterns between these groups were observed in the distribution of EMT scores and the expression heatmaps of the five core genes (Figure 2D). ROC curves evaluated at 1-, 3-, and 5-year time points confirmed the model’s strong discriminative performance (Figure 2E). Kaplan–Meier analysis showed that patients with high EMT scores exhibited significantly higher rates of biochemical recurrence compared to patients with low EMT scores (*p* < 0.0001), confirming that the EMT infiltration score effectively predicts early recurrence risk (Figure 2F,G).

These results indicate significant prognostic differences between risk groups, underscoring the potential clinical utility of the EMT infiltration scoring model for predicting early recurrence in PCa.

### 2.3. Immune Infiltration and Clinical Features of the EMT Infiltration Scoring Model

**Figure 3:**
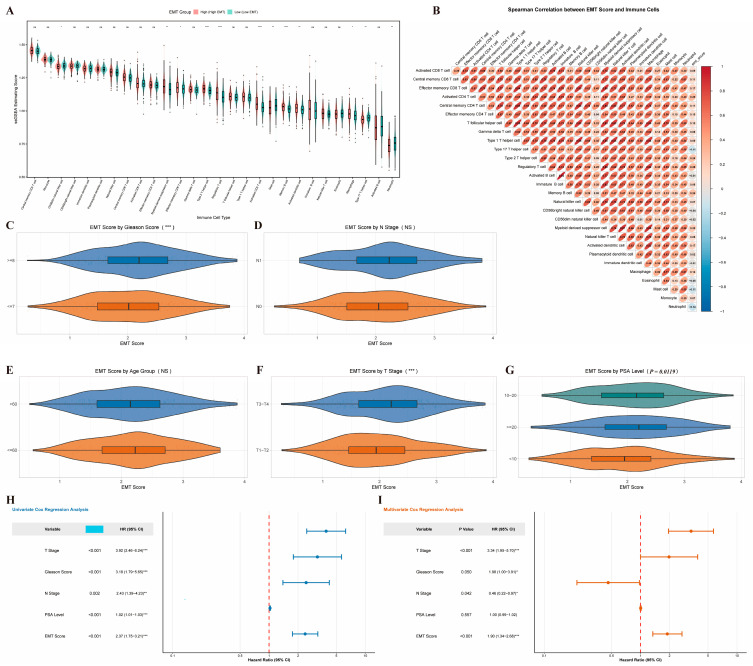
Association analysis of EMT infiltration score with immune and clinical features. (**A**) Differential analysis of 28 immune cell infiltration levels between high- and low-risk groups. (**B**) Spearman correlation analysis between EMT infiltration score and 28 immune cell infiltration levels. (**C**–**G**) Comparison of clinical feature distributions between high- and low-risk groups, including Gleason score (**C**), N stage (**D**), age (**E**), pT stage (**F**), and PSA level (**G**). (**H**) Forest plot of univariate Cox regression analysis for clinical variables and risk score. (**I**) Forest plot of multivariate Cox regression analysis for clinical variables and risk score. * *p* < 0.05, ** *p* < 0.01, *** *p* < 0.001, **** *p* < 0.0001. NS, not significant.

To investigate the impact of EMT on the tumor immune microenvironment, we assessed immune cell composition under varying EMT infiltration levels and determined the associations between EMT infiltration scores and diverse immune cell subsets. Nine immune cell types showed significant differences between patients with high versus low EMT scores determined by ssGSEA. Patients with higher EMT scores exhibited preferential enrichment of multiple immunosuppressive and immunoregulatory populations, comprising CD4^+^ T cells, Tregs, memory B cells (MBCs), myeloid-derived suppressor cells (MDSCs), activated dendritic cells (aDCs), and type 2 helper T cells (Th2). In contrast, neutrophils, mast cells, and type 17 helper T cells (Th17) were relatively more abundant in patients with lower EMT scores (Figure 3A). The data support a relationship between elevated EMT states and a tumor immune microenvironment with heightened complexity and immunosuppressive features.

Correlation analysis further assessed the continuous relationship between EMT infiltration scores and immune cell infiltration levels. After stringent false-discovery rate (FDR < 0.05) correction, 14 immune cell types remained significantly correlated. Activated CD4^+^ T cells, Tregs, Th2 cells, memory B cells, and MDSCs were positively correlated with EMT scores, whereas neutrophils, mast cells, and Th17 cells were negatively correlated (Figure 3B). Among these, activated CD4^+^ T cells and Tregs exhibited the strongest correlation with EMT scores, suggesting that enhanced EMT activity may be closely linked to the recruitment of immunoregulatory T cells [15].

Integrating the differential and correlation analyses, elevated EMT infiltration scores were associated not only with systematic changes in immune cell composition but also with the enrichment of immunosuppressive cell types. This immunological pattern could account for the increased aggressiveness and propensity for early recurrence of prostate cancer linked to EMT.

The association between EMT scores and key clinical parameters—including age, N stage, pT stage, Gleason score, and PSA level—was assessed. EMT scores were notably higher in patients with increased Gleason scores and advanced pT stages (*p* < 0.001). Moreover, significant variations were observed across PSA-level subgroups (*p* = 0.012), suggesting a potential relationship with tumor burden and disease progression. Age and N stage did not show significant associations (Figure 3C–G).

The clinical relevance of the EMT infiltration score for predicting prognosis was examined using univariate and multivariate Cox regression models. Univariate analysis indicated that pT stage, Gleason score, N stage, PSA level, and EMT score were significantly correlated with BCR (Figure 3H). Higher EMT scores significantly increased the risk of BCR (*p* < 0.001). After controlling for these clinical covariates in the multivariate model, the EMT score remained an independent predictor (Figure 3I), whereas PSA lost statistical significance. These results indicate that the EMT infiltration score provides additional prognostic information beyond traditional clinical parameters, supporting its independent predictive value.

### 2.4. Analysis of the Mutational Landscape

EMT is recognized as a key process driving tumor progression and invasiveness. Prior studies have reported that EMT activation frequently coincides with heightened genomic instability and the accumulation of mutations in essential driver genes [16]. Tumor mutational burden (TMB) and specific somatic mutations have also been widely associated with remodeling of the tumor immune microenvironment and immune evasion, potentially affecting response to immunotherapy [17]. To investigate the molecular underpinnings and immunological relevance of EMT infiltration, we examined associations between EMT scores, TMB, and the somatic mutation landscape.

**Figure 4:**
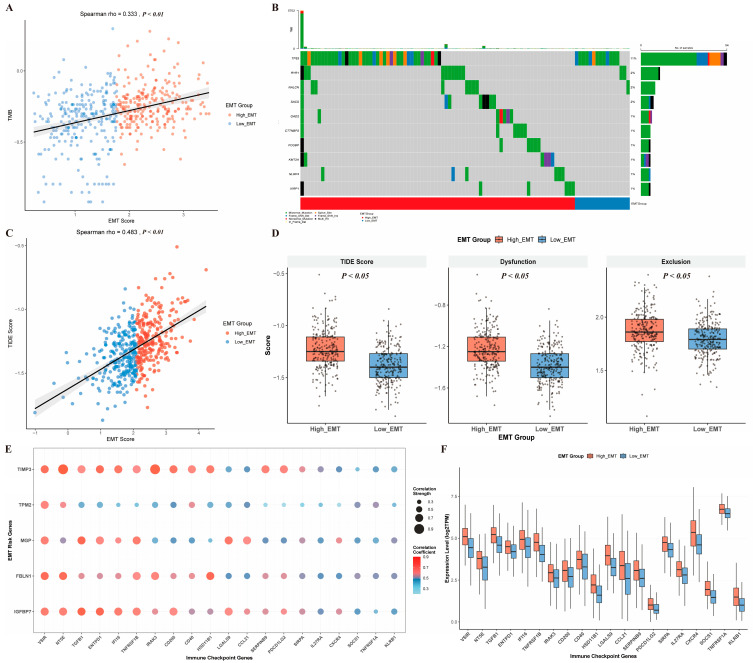
Association analysis of EMT infiltration score with mutation features and immunotherapy-related indicators. (**A**) Spearman correlation analysis between TMB level and EMT infiltration score. (**B**) Mutation waterfall plot of patients with different EMT infiltration score groups, showing the distribution of major mutated genes. (**C**) Spearman correlation analysis between TIDE score and EMT infiltration score. (**D**) Comparison of TIDE score, T cell dysfunction score, and T cell exclusion score between different EMT infiltration score groups. (**E**) Correlation analysis between model risk genes and immune checkpoint gene expression. (**F**) Comparison of immune checkpoint–related gene expression levels between different EMT infiltration score groups.

Spearman correlation analysis revealed a significant positive correlation between EMT scores and TMB (*p* < 0.01), suggesting that enhanced EMT activity may be associated with higher genomic mutation load. This indicates that high EMT tumors may exhibit pronounced genomic instability (Figure 4A).

Analysis of somatic mutations in patients with high versus low EMT scores revealed 10 genes with significantly altered mutation frequencies (*p* < 0.05). Visualization via waterfall plots demonstrated that these mutations were mainly concentrated in the high-EMT group, implying a possible connection between high EMT activity and certain driver mutations (Figure 4B).

Among these, TP53 mutations were most prominent, present in 16.8% of high-scoring EMT patients versus 5.3% of low-scoring patients (Supplementary Figure S4D). As a key tumor suppressor involved in maintaining genomic stability and suppressing invasiveness, TP53 mutations have been frequently linked to EMT activation, enhanced tumor aggressiveness, and unfavorable clinical outcomes [18]. The enrichment of TP53 mutations in high-EMT tumors provides genomic-level support for the association between EMT infiltration and aggressive tumor behavior, further corroborating the molecular heterogeneity reflected by the EMT score.

### 2.5. EMT Infiltration Score and Prediction of Immunotherapy Response

Given that high EMT infiltration scores were associated with enrichment of immunosuppressive cells, increased genomic instability, and accumulation of key driver mutations—features potentially influencing tumor response to immunotherapy—we further evaluated the predictive value of EMT scores for immunotherapy response.

We applied the TIDE algorithm to systematically assess the immune escape characteristics of tumors. TIDE integrates two key immune evasion mechanisms—T cell dysfunction and T cell exclusion—to predict response to immune checkpoint inhibitors [19]. Higher TIDE scores typically indicate poorer responsiveness to immunotherapy.

In our study, EMT scores were significantly positively correlated with overall TIDE scores, suggesting that tumors with enhanced EMT activity may exhibit a more pronounced immune escape phenotype (Figure 4C). Patients with elevated EMT scores exhibited higher overall TIDE scores accompanied by elevated T cell dysfunction and exclusion metrics, suggesting a potential link between EMT infiltration and immune escape–related features (Figure 4D).

Since TIDE only reflects a composite immune escape phenotype, the underlying molecular regulatory mechanisms remain to be explored. Considering the pivotal role of immune checkpoint molecules in regulating T cell suppression and immune tolerance, we assessed the association between EMT scores and the expression of immune checkpoint genes [20].

Correlation analysis was performed between classical immune checkpoint genes and the five core EMT model genes. The mean correlation coefficient across the five risk genes was used as a composite metric to rank immune checkpoint genes. The top 20 immune checkpoint genes most significantly associated with EMT features were visualized using a correlation bubble plot (Figure 4E). These genes were overall positively correlated with EMT model core genes, including several immunosuppressive molecules such as VSIR, NT5E, ENTPD1, LGALS9, and TGFB1, suggesting that EMT activation may be accompanied by coordinated upregulation of immunosuppressive checkpoint pathways.

Further analysis revealed that, among patients stratified by EMT scores, immune checkpoint genes were largely upregulated in patients with higher EMT scores (Figure 4F). Boxplot analysis confirmed significant differences for most immune checkpoint molecules, consistent with their correlation with EMT risk genes. These results provide molecular-level evidence that EMT infiltration is closely linked with activation of immunosuppressive checkpoint pathways.

### 2.6. Prognostic Value Assessment of Core EMT Genes

Building on the demonstrated prognostic value of the EMT infiltration score, we further explored the expression patterns of the five core genes and their associations with EMT activity and BCR outcomes.

**Figure 5:**
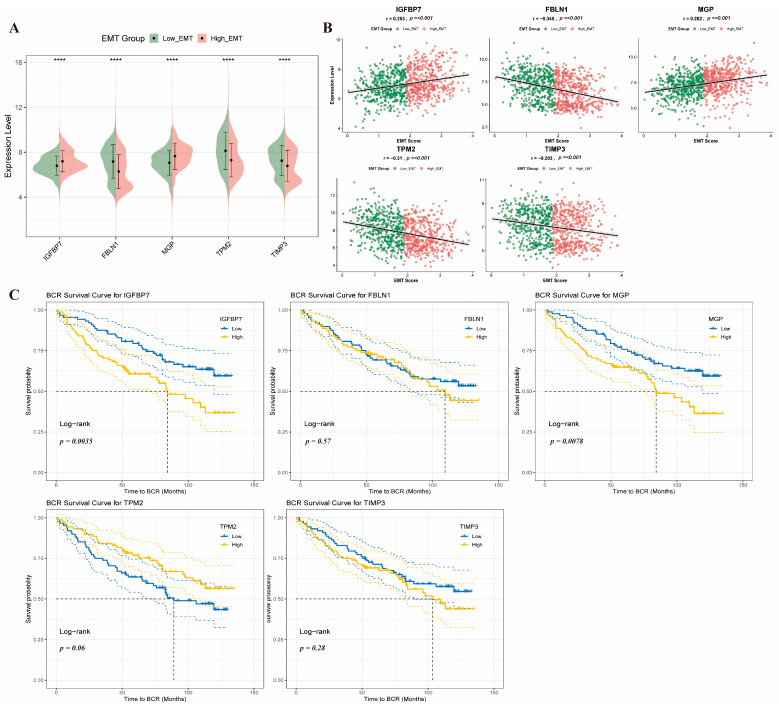
Expression characteristics and prognostic value of five core EMT-related genes. (**A**) Comparison of expression levels of the five core EMT-related genes between high and low EMT score groups. Statistical significance was determined by *t*-test: **** *p* < 0.0001, NS, not significant. (**B**) Spearman correlation analysis between expression levels of the five core EMT-related genes and EMT infiltration score. (**C**) Kaplan-Meier survival curves for BCR-free survival stratified by high (red) and low (green) expression of each gene based on median expression cut-off. The dashed horizontal and vertical lines indicate the median survival time for each group. *p*-values were calculated using the log-rank test.

Comparison of gene expression between patients with high versus low EMT scores revealed that IGFBP7 and MGP were significantly upregulated in high-scoring patients, whereas FBLN1, TPM2, and TIMP3 were predominantly expressed in low-scoring patients (Figure 5A). This indicates that the core genes exhibit directionally consistent yet functionally distinct expression patterns across EMT-defined subgroups.

Correlation analysis further confirmed that IGFBP7 and MGP expression levels were positively correlated with EMT infiltration scores, while FBLN1, TPM2, and TIMP3 showed significant negative correlations (Figure 5B). These directional trends are consistent with the expression patterns observed in high- and low-EMT groups, supporting the potential biological relevance of these genes in regulating EMT activity.

We then systematically evaluated the prognostic value of the five core genes in the external validation cohort GSE220095. Patients were stratified into high- and low-expression groups based on the median expression of each gene, and Kaplan–Meier survival analysis was performed with BCR as the endpoint (Figure 5C). High expression of IGFBP7 and MGP was associated with a significantly increased risk of BCR, suggesting that these genes may serve as key risk factors promoting EMT-related invasive phenotypes and early recurrence. In contrast, FBLN1, TPM2, and TIMP3 did not reach statistical significance in single-gene survival analysis, although TPM2 high-expression patients tended to show reduced BCR risk. These results indicate that while some core genes may have limited predictive power individually, their combined effect in a multi-gene model remains highly informative.

Overall, the five core genes collectively form a robust EMT infiltration scoring model, providing gene-level support for the biological rationale and clinical potential of the EMT score as a tool for assessing early recurrence risk in PCa.

### 2.7. Pathway Enrichment Analysis

To investigate the potential biological processes underlying different EMT infiltration states, we performed gene set enrichment analysis (GSEA). Based on the EMT infiltration scores, prostate cancer patients were divided into subgroups with relatively higher or lower EMT activity. Subsequent genome-wide comparison of gene expression between these subgroups identified 13,266 significantly differentially expressed genes (FDR < 0.05), with 10,719 upregulated in the high-EMT group and 2547 upregulated in the low-EMT group (Supplementary Figure S4E).

Enrichment analysis of the differential gene set identified 38 pathways with significant variation across EMT infiltration score–stratified patients (FDR < 0.05), presented in a bubble plot (Supplementary Figure S5). These pathways encompass multiple biological dimensions, including signal transduction, cell–cell communication, immunoregulation, and cellular structural composition, suggesting that changes in EMT infiltration status are accompanied by multidimensional functional remodeling.

**Figure 6:**
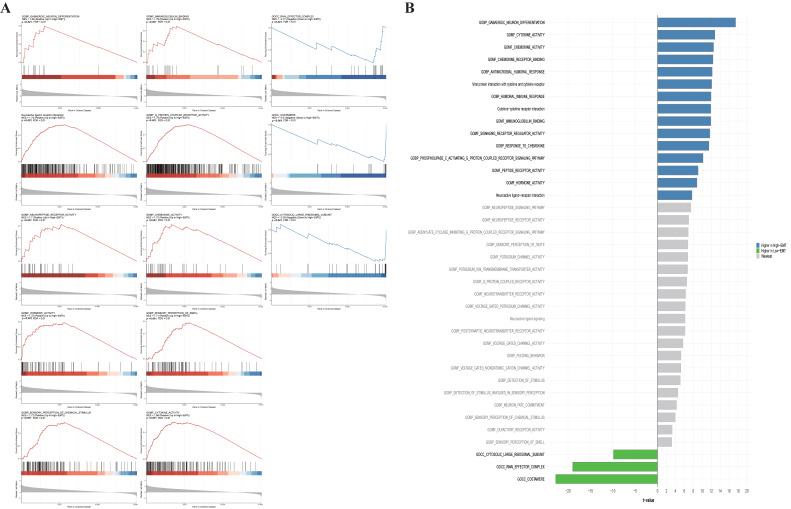
Functional pathway enrichment analysis of different EMT infiltration score groups. (**A**) Functional pathway enrichment results based on GSEA, showing biological pathways with significant intergroup differences. (**B**) GSVA analysis results, depicting differences in activity levels of various functional pathways between groups.

Considering the known biological roles of EMT in tumor invasion and immune modulation, 13 representative pathways closely related to EMT processes were selected from the significantly differential pathways and visualized using enrichment curves (Figure 6A). Functional enrichment comparisons indicated divergent biological programs between EMT infiltration score–defined patient groups. EMT score–elevated samples showed dominant activation of signaling, intercellular communication, and immune-associated pathways, whereas EMT score–reduced samples were preferentially enriched in structural maintenance and protein translation processes, such as RNA interference effector complexes and cytoplasmic ribosomal large subunits.

To assess pathway activity at the sample level, the 38 significantly differential pathways were subjected to gene set variation analysis (GSVA), and GSVA scores were compared between high- and low-EMT groups. Heatmap visualization indicated clear stratification of pathway activity between patients with high versus low EMT scores (Supplementary Figure S6). Differential GSVA scores were further visualized in bar plots (Figure 6B), with pathways showing the largest absolute t-values highlighted to emphasize significant differences.

Overall, most pathways exhibited positive t-values in patients with high EMT scores, primarily involving cytokine–receptor interactions, chemokine activity, humoral immune response, and neuropeptide-related signaling. In contrast, a minority of pathways were relatively enriched in patients with low EMT scores, including costamere structures, RNAi effector complexes, and cytoplasmic ribosomal large subunits, reflecting preservation of cellular structural stability and protein translation functions.

These results demonstrate that tumors with distinct EMT infiltration states exhibit significant functional pathway differences, with high-EMT states favoring activation of signaling and immune-related pathways, while low-EMT states retain baseline structural and metabolic functions.

### 2.8. Targeted Drug Screening

In this study, molecular docking analyses were performed focusing on the five core EMT-related proteins identified in patients with high EMT scores to evaluate their predicted interactions with known drug molecules.

**Figure 7:**
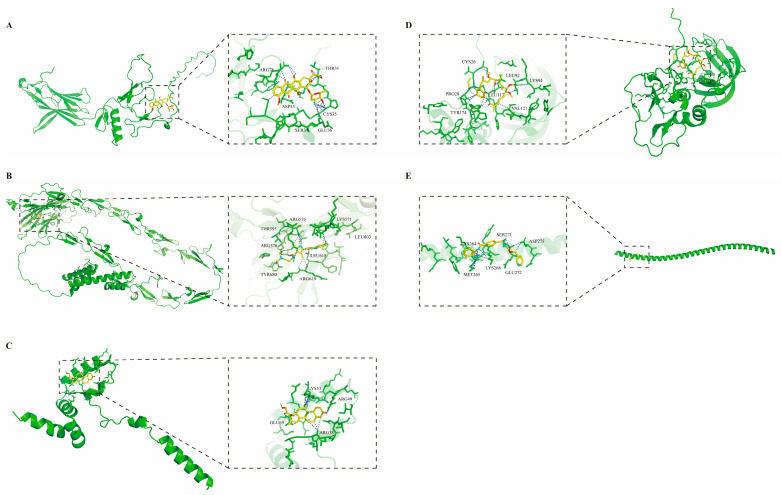
Molecular docking results between core EMT-related proteins and candidate drugs. (**A**) Molecular docking mode of IGFBP7 with Doxorubicin. (**B**) Molecular docking mode of FBLN1 with Sunitinib. (**C**) Molecular docking mode of MGP with Dexamethasone. (**D**) Molecular docking mode of TPM2 with Entinostat. (**E**) Molecular docking mode of TIMP3 with Tanespimycin.

Computational docking suggested a potential protein–ligand interaction between IGFBP7 and doxorubicin, with a predicted binding energy of −6.8 kcal/mol (Figure 7A). Doxorubicin is a classical anthracycline chemotherapeutic agent that primarily acts by intercalating into the DNA double helix and inhibiting topoisomerase II activity, thereby blocking tumor cell proliferation. The compound has been broadly applied in clinical oncology for the management of diverse solid cancers [21].

Docking simulations for FBLN1 suggested a predicted binding energy of −6.8 kcal/mol with sunitinib (Figure 7B). Sunitinib is a multi-targeted tyrosine kinase inhibitor that acts on multiple pathways including VEGFR and PDGFR, and has been reported to exhibit antitumor and antiangiogenic activities in cancer studies [22].

In the molecular docking analysis targeting MGP, dexamethasone displayed a putative interaction, with a predicted binding energy of −7.1 kcal/mol (Figure 7C). Dexamethasone is a commonly used glucocorticoid with anti-inflammatory and immunomodulatory properties, and is commonly used in cancer therapy to alleviate inflammation and reduce treatment-related adverse effects [23].

Molecular docking suggested a potential interaction pattern between TIMP3 and tanespimycin, with a predicted binding energy of −7.3 kcal/mol (Figure 7D). Tanespimycin is an Hsp90 inhibitor that has been reported to disrupt the stability of multiple oncogenic proteins and has been studied in various cancer contexts [24].

Finally, molecular docking of TPM2 with entinostat indicated a predicted binding energy of −6.1 kcal/mol (Figure 7E). Entinostat, a selective histone deacetylase (HDAC) inhibitor, has been reported to modulate chromatin structure and gene transcription, thereby affecting tumor cell growth and differentiation [25].

As negative controls, molecular docking simulations using small compounds showed markedly lower binding affinities compared to the therapeutic candidates (Supplementary Figure S7), supporting the specificity of the predicted protein–drug interactions.

## 3. Discussion

PCa is one of the most prevalent malignancies among men worldwide. Although the majority of patients respond well to early interventions such as surgery, radiotherapy, or endocrine therapy, disease recurrence and progression remain major challenges affecting long-term survival. Traditional treatment strategies still have limitations in effectively suppressing tumor invasion and metastasis. Increasing evidence indicates that EMT, a key biological process driving enhanced tumor invasiveness and metastatic potential, plays an essential role in PCa progression. With the advent of scRNA-seq technology, researchers are now able to resolve intra-tumor heterogeneity and functional state transitions at single-cell resolution, providing powerful tools for accurately characterizing EMT features and constructing biologically grounded predictive models.

In this study, we first systematically analyzed the cellular composition of PCa tissues based on multi-cohort scRNA-seq data. After rigorous quality control and batch effect correction, various cell populations, including epithelial, immune, and stromal cells, were identified, and epithelial lineage cells were isolated for downstream analyses. Previous studies have shown that tumor epithelial cells are the core population driving PCa invasion and metastasis, and their transcriptional heterogeneity is closely associated with disease progression [26]. Focusing on malignant epithelial cells thus enables a more precise characterization of EMT-associated molecular features.

Next, we employed the CopyKAT algorithm to identify malignant epithelial cells based on CNV profiles, allowing the distinction between tumor-derived and normal epithelial cells at the single-cell level. Notably, EMT-related transcriptional reprogramming often involves not only changes in transcriptional regulatory networks but also genomic instability, including CNV accumulation and chromosomal rearrangements. These alterations are believed to enhance phenotypic plasticity and enable cells to better adapt to microenvironmental pressures. Identifying malignant cells based on CNV profiles not only reflects genomic instability but may also indirectly indicate their potential for EMT-associated transcriptional reprogramming [27]. In our study, the distinct distributions of cells with different CNV states in low-dimensional space further validated the transcriptional heterogeneity between malignant and normal cells, providing a solid foundation for subsequent EMT state analyses.

After identifying malignant epithelial cells, we quantified EMT activity at the single-cell level using the AUCell algorithm based on EMT-related gene sets from the MSigDB database [28]. Malignant cells were stratified into EMT-high and EMT-low groups, facilitating the identification of key transcriptional features associated with invasive phenotypes. Differential expression analysis between these groups allowed us to select candidate genes significantly correlated with EMT activity, and ssGSEA further assessed their functional relevance at the pathway level. This process ultimately identified 16 core EMT-related genes for subsequent model construction. Previous studies have demonstrated that EMT is a complex biological process driven by multiple signaling pathways and transcriptional regulatory networks, and integrating multi-level transcriptional information enhances the accurate capture of EMT-associated molecular features [29].

To improve the prognostic value of current clinicopathological factors [30], we integrated multi-cohort bulk transcriptomic data to construct an EMT infiltration scoring model. Using LASSO regression, five key genes were selected, enabling effective quantification of tumor EMT activity and prediction of BCR risk. The EMT infiltration scoring model fundamentally differs from conventional T staging in assessing tumor invasiveness. While T staging reflects macroscopic tumor burden based on anatomical extent and local invasion [31], the EMT infiltration score captures the transcriptional activity of EMT-related tumor phenotypes. Thus, the score complements rather than replaces T staging, providing molecular-level insights into tumor aggressiveness.

PCa is generally considered an immunologically “cold” tumor, characterized by low effector T cell infiltration, a relatively immunosuppressive environment, and weak inflammatory responses, which is consistent with the limited efficacy of immune checkpoint inhibitors in PCa [32]. Typical immune features in PCa include low CD8^+^ T cell infiltration, Tregs, MDSCs, and immunosuppressive cytokine expression. These factors collectively establish an inhibitory immune microenvironment associated with tumor progression and therapy resistance [33]. Prior studies have also demonstrated that EMT activation in PCa is closely linked to malignant progression, immune microenvironment dysregulation, and increased BCR risk. Patients with high expression of EMT-related molecules are more prone to develop an immunosuppressive microenvironment following BCR, further accelerating tumor progression [34], providing the theoretical basis for our study on EMT-mediated immune remodeling in PCa.

Changes in immune features among high EMT infiltration patients are partially consistent with PCa’s inherent immunosuppressive state but also exhibit EMT-specific patterns. In high-EMT patients, Tregs, MDSCs, activated CD4^+^ T cells, Th2 cells, and activated dendritic cells were significantly enriched and positively correlated with EMT scores. Tregs and MDSCs are core immunosuppressive populations: Tregs secrete IL-10 and TGF-β to directly inhibit effector T cell function, while MDSCs utilize ARG1 and iNOS pathways to promote and maintain an immunosuppressive microenvironment, facilitating tumor invasion and progression [35]. Additionally, the enrichment of activated CD4^+^ T cells and Th2 cells in the high EMT group does not reflect effective antitumor immunity, as Th2 cells preferentially secrete tissue remodeling factors such as IL-4 and IL-13 rather than enhancing cytotoxic immune responses [36]. Dysfunctional dendritic cells, despite increased numbers, exhibit impaired antigen presentation, collectively exacerbating immune tolerance [37]. Conversely, antitumor immune cells such as neutrophils, mast cells, and Th17 cells were significantly reduced in high EMT patients, consistent with prior reports on EMT-associated immunosuppression. In particular, Th17 cells, which can contribute to antitumor immunity, are often suppressed in enhanced EMT contexts [38].

Considering that EMT may promote genomic instability, we analyzed mutation profiles in high- versus low-EMT patients. High EMT scores were associated with increased overall TMB, suggesting that EMT may drive genomic instability in PCa, making key tumor suppressor genes such as TP53 more susceptible to mutation. TP53 mutations are known to contribute to PCa progression and can modulate the secretion of immunoregulatory factors, influencing MDSCs, tumor-associated macrophages, and suppressing antitumor Th17 responses, thereby intensifying the intrinsic immunosuppressive state of PCa [39]. These findings indicate that EMT not only directly reshapes immune cell composition but also indirectly exacerbates immunosuppressive features via genomic instability, creating a dual mechanism for immune evasion.

Tumor immune evasion is a critical mechanism underlying cancer progression and immunotherapy resistance. TIDE scoring assesses the potential of tumors to escape immune surveillance. In our analysis, high-EMT patients exhibited significantly elevated TIDE scores, indicating potential suppression of effector T cell function and increased activity of regulatory or immunosuppressive cells. This aligns with prior immune infiltration analyses, further supporting that EMT-high tumors manifest an overall immunosuppressive microenvironment.

Immune checkpoints suppress effector T cell activity or enhance regulatory immune cell function, facilitating tumor immune evasion. Although PCa is considered immunologically cold, molecules such as PD-1/PD-L1, CTLA-4, and newer inhibitory molecules including VSIR and NT5E remain active within the PCa microenvironment, contributing to limited immunotherapy efficacy [40]. In high EMT patients, multiple key immune checkpoint molecules were significantly upregulated and positively correlated with the five core EMT risk genes. For example, VSIR, NT5E, and TGFB1 may inhibit CD8^+^ T cell activity, enhance regulatory immune cell function, and remodel the tumor microenvironment, reinforcing immune evasion [41].

Pathway enrichment analyses (GSEA and GSVA) further revealed functional differences between high- and low-EMT groups: high-EMT tumors were enriched for immune signaling, cytokine–receptor interactions, and chemokine pathways, whereas low-EMT tumors were enriched in pathways related to cellular structural maintenance and protein synthesis. These results confirm that EMT not only alters immune cell composition but also reshapes molecular signaling networks, creating a microenvironment favorable for tumor invasion and immune escape. Collectively, multi-dimensional analyses indicate that EMT acts as a core regulator of tumor immune microenvironment remodeling, promoting PCa progression through associations with genomic instability, accumulation of immunosuppressive cells, and activation of immune checkpoints [42].

Furthermore, this study focused on five core EMT risk genes: IGFBP7, FBLN1, MGP, TPM2, and TIMP3. IGFBP7 regulates proliferation and apoptosis in various solid tumors and is associated with immunomodulation in the tumor microenvironment, potentially inhibiting tumor cell proliferation and migration via MAPK/ERK and PI3K/AKT signaling [43]. FBLN1, an extracellular matrix protein, contributes to matrix remodeling and cell adhesion, primarily through TGF-β/SMAD signaling, enhancing tumor invasiveness [44]. MGP is involved in calcium metabolism and signaling, influencing BMP/SMAD and NF-κB pathways while modulating tumor immune contexture [45]. TPM2 regulates the actin cytoskeleton and participates in Rho/ROCK signaling to control EMT phenotype and cell migration [46]. TIMP3, a matrix metalloproteinase inhibitor, stabilizes the ECM via ADAMs/Hsp90 pathways and regulates signaling molecule release, impacting tumor progression and immunoregulation [47]. These biological functions align with our findings linking EMT activity to immunosuppressive microenvironments.

Molecular docking analyses identified putative binding interactions. IGFBP7 exhibited binding affinity toward doxorubicin, a chemotherapeutic agent known to induce DNA damage and trigger apoptosis, supporting a plausible chemical–protein interaction. FBLN1 bound sunitinib, a multi-target tyrosine kinase inhibitor targeting VEGFR and PDGFR signaling pathways, which indicates a potential interaction relevant to tumor-related signaling processes. MGP interacted with dexamethasone, a glucocorticoid involved in modulation of inflammatory responses and immune activity, suggesting an interaction with immune-related compounds. TPM2 demonstrated docking with entinostat, an HDAC inhibitor affecting cell cycle, apoptosis, and differentiation, suggesting a regulatory-level interaction. TIMP3 bound tanespimycin, an Hsp90 inhibitor destabilizing multiple signaling proteins, which is associated with ECM-related processes. These docking results suggest potential binding relationships between candidate genes and compounds, which may provide supportive computational evidence for hypothesis generation in high-risk PCa, although no direct therapeutic conclusions can be drawn. Experimental validation is required to confirm these interactions.

In summary, we integrated scRNA-seq and multi-cohort transcriptomic data and performed quality control, cell annotation, malignant epithelial cell identification, and EMT scoring to stratify high- and low-EMT populations, identifying core EMT-related genes. Based on these, an EMT infiltration scoring model was constructed and validated in external cohorts, analyzing its correlations with immune infiltration, mutation features, and clinical outcomes, and predicting potential therapeutic drugs via molecular docking.

However, several limitations exist. First, analyses were primarily computational, and the protein-level expression and functional roles of core genes have not yet been validated experimentally. Second, model construction and validation relied on public cohorts, lacking prospective clinical samples, particularly with complete BCR follow-up, which limits generalizability.

Future directions include validating core gene expression and effects on EMT activity, proliferation, migration, and invasion in PCa cell models, evaluating their roles and potential drug interventions in mouse PCa models, and integrating single-cell and spatial omics to further delineate mechanisms of core genes in the tumor immune microenvironment and their relationship with EMT states.

In conclusion, this study systematically elucidates EMT activity and associated immune microenvironment features in PCa from single-cell to population levels and identifies five core genes and potential therapeutic targets, providing a theoretical basis and new insights for predicting PCa invasiveness and enabling personalized treatment strategies.

## 4. Materials and Methods

### 4.1. Study Workflow

**Figure 8:**
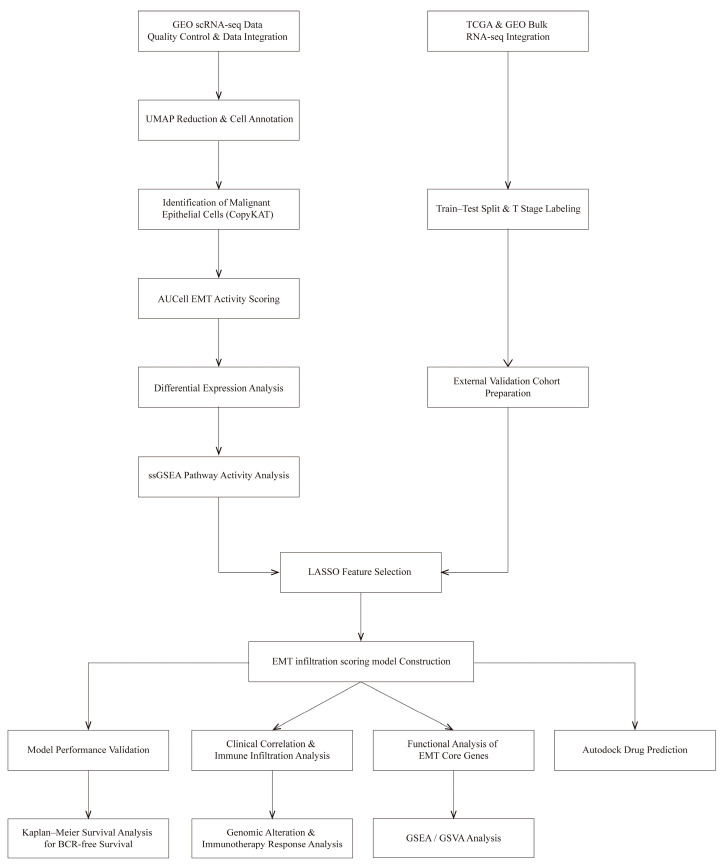
Overview of study design and analysis workflow. This figure illustrates the steps involved in the study, including sample collection, data preprocessing, selection of EMT-related genes, and model construction and validation.

The overall workflow of this study is illustrated in Figure 8, summarizing the study design, including sample collection, data preprocessing and normalization, predictive model development, subsequent validation procedures, immune infiltration and molecular function analyses, and molecular docking-based prediction of potential therapeutic compounds.

### 4.2. Data Sources and Preprocessing

The scRNA-seq data used in this study were obtained from the GEO database [48], including GSE141445, GSE181294, and GSE193337. GSE141445 contains 13 PCa tumor samples, GSE181294 includes 20 PCa tumor samples, and GSE193337 comprises malignant tumor samples from 4 patients. Bulk RNA-seq data were derived from the CGA-PRAD cohort in the CGA database and the GEO datasets GSE62116 and GSE220095 [49]. The CGA-PRAD and GSE62116 datasets were used for model training and internal validation, while GSE220095 served as an independent external validation cohort to assess model generalizability. All data were obtained from public repositories and complied with data-sharing and ethical regulations.

### 4.3. Single-Cell RNA-Seq Data Processing and Quality Control

scRNA-seq data analysis was performed using R (v4.3.2) and the Seurat package (v4.4.0). Data from different samples were integrated and normalized, with batch effects corrected using standard pipelines. Cells were filtered based on the following quality criteria: (1) 300 < nFeature_RNA < 6000; (2) percent.mt ≤ 15%; (3) 200 < nCount_RNA < 40,000. Highly variable genes were selected for downstream analysis, followed by principal component analysis (PCA) for preliminary dimensionality reduction. Uniform manifold approximation and projection (UMAP) was used for nonlinear dimensionality reduction and visualization. Unsupervised clustering was performed using a nearest-neighbor approach. Cell-type annotation was conducted based on canonical marker gene expression and reference databases, including SingleR (v2.4.1), CellMarker, and PanglaoDB. Major cell populations were identified, including epithelial cells, immune cells, and stromal cells [50]. CopyKAT (v1.1.0) was applied to infer CNV patterns, allowing distinction between tumor-derived malignant epithelial cells (aneuploid) and normal epithelial cells (diploid) based on genome-wide CNV profiles.

### 4.4. EMT Activity Assessment, Grouping, and Differential Gene Screening

EMT activity in single cells was quantified using the AUCell algorithm, which calculates the AUC for a gene set based on expression rankings, reflecting its activity in each cell [51]. EMT-related gene sets were obtained from the MSigDB database using the “HALLMARK_EPITHELIAL_MESENCHYMAL_TRANSITION” gene set, and were used to compute AUC scores for each malignant epithelial cell. Cells were stratified into EMT-high and EMT-low groups based on the median AUC score. The resulting groups were visualized in conjunction with dimensionality reduction results. Differential expression analysis between EMT-high and EMT-low cells was performed, with multiple testing correction using the Benjamini–Hochberg method. Genes with an adjusted *p*-value (*p*adj) < 0.01 and |avg_log2FC| > 0.5 were considered significantly differentially expressed. Furthermore, ssGSEA was applied to quantify gene set activity at the single-cell or sample level, allowing selection of candidate genes with high correlation to EMT status for downstream analyses.

### 4.5. Construction of the EMT Infiltration Score Model

Candidate genes significantly associated with EMT activity were used to construct the EMT infiltration scoring model. The CGA-PRAD cohort (n = 502) and GSE62116 dataset (n = 235) were used as the training set. Expression matrices were merged, and batch effects were corrected using the ComBat algorithm in the sva R package (v3.50.0). GSE220095 (n = 176) was used as an independent external validation cohort for model evaluation. Model construction was implemented in R using the glmnet package (v4.1-8) with LASSO logistic regression for feature selection and model building. An L1 regularization term was incorporated to shrink gene coefficients, reducing model complexity and mitigating multicollinearity [52]. Tenfold cross-validation was performed to optimize the penalty parameter λ, using binomial deviance as the performance metric. Pathological T stage (pT stage) information was used as clinical annotation for survival and risk stratification analyses. The optimal λ was selected to determine the final set of core genes included in the model.

To evaluate the prognostic relevance of core genes, univariate and multivariate Cox proportional hazard regression analyses were performed to calculate the hazard ratios (HRs) and 95% confidence intervals (CIs), and corresponding *p*-values reported. Time-dependent ROC analysis was applied to assess model performance, with the AUC and corresponding 95% confidence intervals (CIs) computed for each cohort [53]. In the external validation cohort, patients were stratified into high-risk and low-risk groups based on EMT infiltration scores. Kaplan–Meier survival analysis was conducted with log-rank tests to assess group differences [54], and *p*-values < 0.05 were considered statistically significant.

To assess clinical utility and individualized risk prediction, a nomogram was constructed using the rms package (v6.7-1), integrating EMT infiltration scores with clinical features [55]. All statistical analyses applied the Benjamini–Hochberg method for multiple testing correction (*p*adj < 0.01).

### 4.6. Immune Infiltration Analysis and Clinical Correlation

ssGSEA was employed to quantify the infiltration levels of 28 immune cell types in the CGA cohort. Spearman rank correlation analysis assessed associations between EMT infiltration scores and immune cell abundance, and corresponding correlation coefficients (ρ) and *p*-values were calculated. Correlation matrices were visualized using the corrplot package (v0.95) for intuitive interpretation. Patient clinical features, including age, pT stage, N stage, Gleason score, and PSA levels, were collected. Differences in EMT infiltration scores across clinical subgroups were evaluated using the Wilcoxon rank-sum test or Kruskal–Wallis test. Univariate and multivariate Cox regression models were applied to examine the relationships between EMT infiltration scores, clinical variables, and BCR risk.

### 4.7. Somatic Mutation and Tumor Mutation Burden Analysis

Somatic mutation data were obtained from TCGA. Mutational analysis and visualization were performed using the maftools package (v2.18.0) [56]. TMB was calculated for each sample, and its correlation with EMT infiltration scores was evaluated. Mutation frequencies of individual genes were compared between high-EMT and low-EMT groups, with waterfall plots illustrating the distribution of major mutated genes.

### 4.8. Immunotherapy Response Prediction

The TIDE algorithm was used to predict potential immune checkpoint inhibitor responses by simulating tumor immune escape mechanisms, integrating T cell dysfunction and T cell exclusion features. TIDE scores, T cell dysfunction scores, and T cell exclusion scores were obtained from the TIDE platform (http://tide.dfci.harvard.edu/, accessed on 10 November 2025) and compared between high-EMT and low-EMT groups. Immune checkpoint-related gene sets were downloaded from the ImmPort database. Correlations between EMT infiltration model core genes and immune checkpoint genes were assessed, and nonparametric tests were used to compare expression differences between EMT groups [57]. Significantly correlated immune checkpoint genes were visualized to characterize potential associations between EMT features and immunoregulatory molecules.

### 4.9. Core Gene Expression and Prognostic Analysis

Expression levels of the five core EMT genes in different risk groups were compared, and Spearman correlation analysis assessed associations with EMT infiltration scores. Patients were stratified into high-expression and low-expression groups based on median gene expression, and Kaplan–Meier survival analysis was performed to evaluate BCR outcomes, with log-rank tests assessing statistical significance. Multiple testing corrections were applied using the Benjamini–Hochberg method (*p*adj < 0.01).

### 4.10. GSEA and GSVA Analysis

GSEA was performed to identify biological pathways significantly enriched between high- and low-EMT-risk groups by evaluating the distribution of predefined gene sets in ranked gene expression lists. GSVA was then applied to quantify pathway activity scores at the individual sample level, enabling statistical comparison and visualization of pathway differences between high-EMT and low-EMT groups. All analyses were performed using the GSVA package (v1.50.5), enabling a comprehensive workflow from group-level enrichment to single-sample pathway activity quantification.

### 4.11. Molecular Docking and Drug Screening

Molecular docking and potential drug screening were conducted using AutoDock (v4.2, Linux). Small-molecule compounds related to prognostic core genes were collected from curated gene–chemical interaction records in the Comparative Toxicogenomics Database (CTD) [58], and their structural information was obtained from PubChem. Protein structures corresponding to core genes were downloaded from UniProt [59]. First, batch molecular docking simulations were performed for all CTD-derived small molecules against the target proteins. Subsequently, compounds were ranked according to binding energy, and candidates with higher binding affinity were selected for downstream analysis, and docking results were visualized using PyMOl (v3.1, open-source). This workflow ensures reproducibility while avoiding redundant data downloads. To ensure robustness of docking interpretation, control compounds were included for comparison, including a representative active reference compound and a structurally unrelated small molecule as a negative control. Binding energies were interpreted in a relative manner rather than as absolute indicators of pharmacological efficacy.

## 5. Conclusions

In this study, we systematically characterized EMT heterogeneity in malignant epithelial cells of PCa at the single-cell level using multi-cohort scRNA-seq and bulk transcriptomic data. We successfully constructed and validated an EMT infiltration scoring model comprising five core genes: IGFBP7, FBLN1, MGP, TPM2, and TIMP3. The model demonstrated robust predictive performance across the internal training set, test set, and an independent GEO external validation cohort. It effectively quantifies tumor EMT activity, accurately stratifies patients by BCR risk, and retains independent prognostic value even after adjustment for conventional clinical indicators, providing a molecular basis for PCa risk stratification that extends beyond anatomical staging.

Mechanistically, our study revealed that high EMT activity is closely associated with remodeling of the PCa immune microenvironment. The high-EMT-score group was enriched for immunosuppressive cells, including Tregs and MDSCs, while antitumor immune cells such as neutrophils and Th17 cells were reduced, forming a characteristic immunosuppressive phenotype. EMT activity was also significantly correlated with TMB and TP53 mutations, suggesting that genomic instability may synergize with EMT to promote tumor progression. Furthermore, high-EMT patients exhibited elevated TIDE scores and upregulation of immune checkpoint genes (e.g., VSIR, NT5E), providing potential biomarkers for predicting immunotherapy efficacy. Pathway enrichment analysis indicated that high-EMT tumors primarily activate immune-related signaling and cytokine–receptor interaction pathways, whereas low-EMT tumors are enriched in pathways related to cellular structural maintenance and protein synthesis. Molecular docking identified potential targeted drugs, such as doxorubicin and sunitinib, offering new therapeutic avenues for high EMT-activity PCa.

In summary, through multi-dimensional analyses, this study elucidates the central regulatory role of EMT in PCa progression. The EMT infiltration scoring model combines biological rationality with clinical applicability, providing an important molecular foundation for precise prognostic assessment, risk stratification, and individualized treatment decisions in PCa. Additionally, the revealed associations between EMT, the immune microenvironment, and genomic instability, along with the identified potential targeted drugs, offer a scientific basis for the development of targeted intervention strategies in future studies.

## Data Availability

All data analyzed in this study were obtained from publicly available databases. Further inquiries can be directed to the corresponding author Zhipeng Xie at 13977391933@163.com.

## Supplementary Materials

The following supporting information can be downloaded at https://www.mdpi.com/article/10.3390/ijms27115017/s1.

## Abbreviations

PCa	Prostate cancer
EMT	Epithelial–mesenchymal transition
scRNA-seq	Single-cell RNA sequencing
ML	Machine learning
CNV	Copy number variation
BCR	Biochemical recurrence
TIME	Tumor immune microenvironment
LASSO	Least absolute shrinkage and selection operator
Survival-SVM	Survival support vector machine
Enet	Elastic net
ICP	Immune checkpoint
RS	Risk stratification
TMB	Tumor mutational burden
MD	Molecular docking
GSEA	Gene set enrichment analysis
TIDE	Tumor immune dysfunction and exclusion
Tregs	Regulatory T cells
MDSCs	Myeloid-derived suppressor cells
IME	Immunosuppressive microenvironment
OS	Overall survival
HPA	Human Protein Atlas
CTD	Comparative Toxicogenomics Database
RMSD	Root-mean-square deviation
RMSF	Root-mean-square fluctuation
ROC	Receiver Operating Characteristic
