# Supplementary material for: Integrated Single-Cell RNA-Seq and Machine Learning to Construct an EMT Infiltration Scoring Model for Prostate Cancer
Source: Int J Mol Sci. 2026 Jun 2;27(11):5017. doi: 10.3390/ijms27115017 (PMC13256516; doi:10.3390/ijms27115017)
Supplement: Supplementary file 1 [file ijms-27-05017-s001.zip › ijms-4260190-supplementary.pdf]

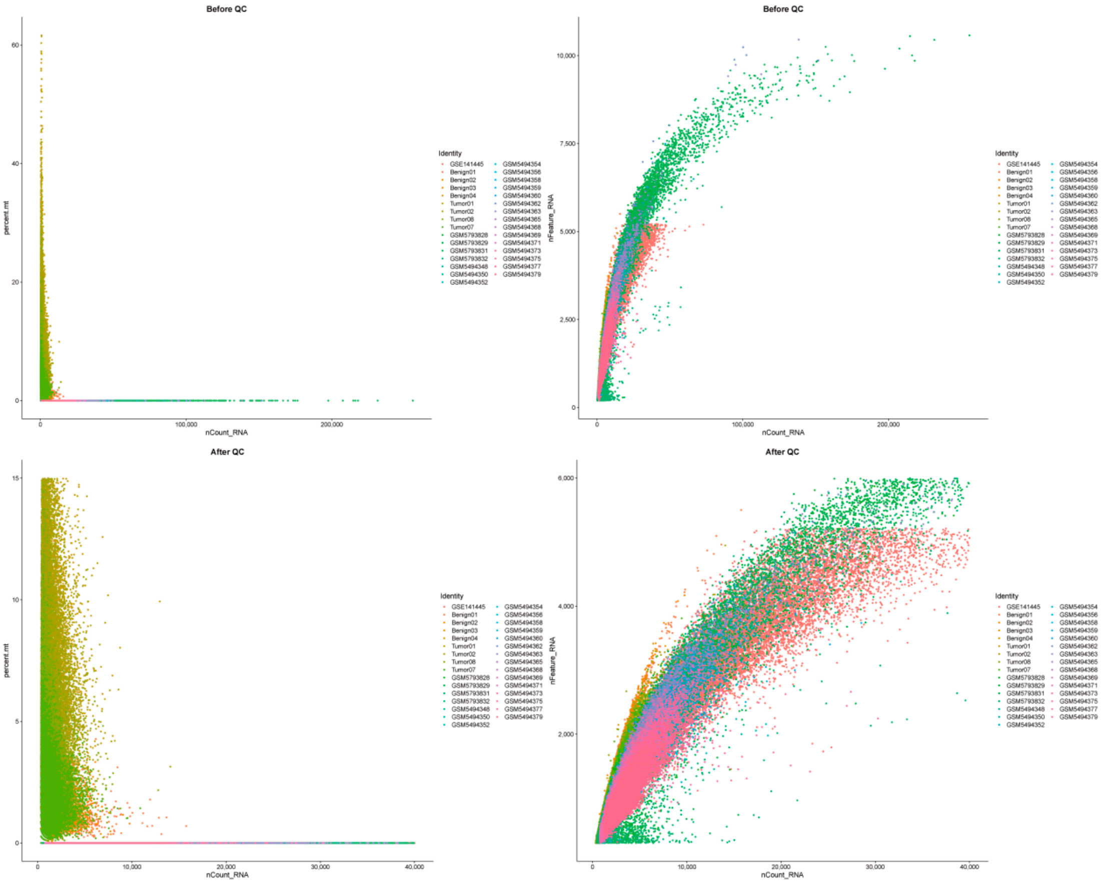

Supplementary Figure S1. Quality control metrics for single-cell transcriptome data. Scatter plots for single-cell transcriptome data quality control, showing distributions of gene counts, UMI counts, and mitochondrial gene proportion.

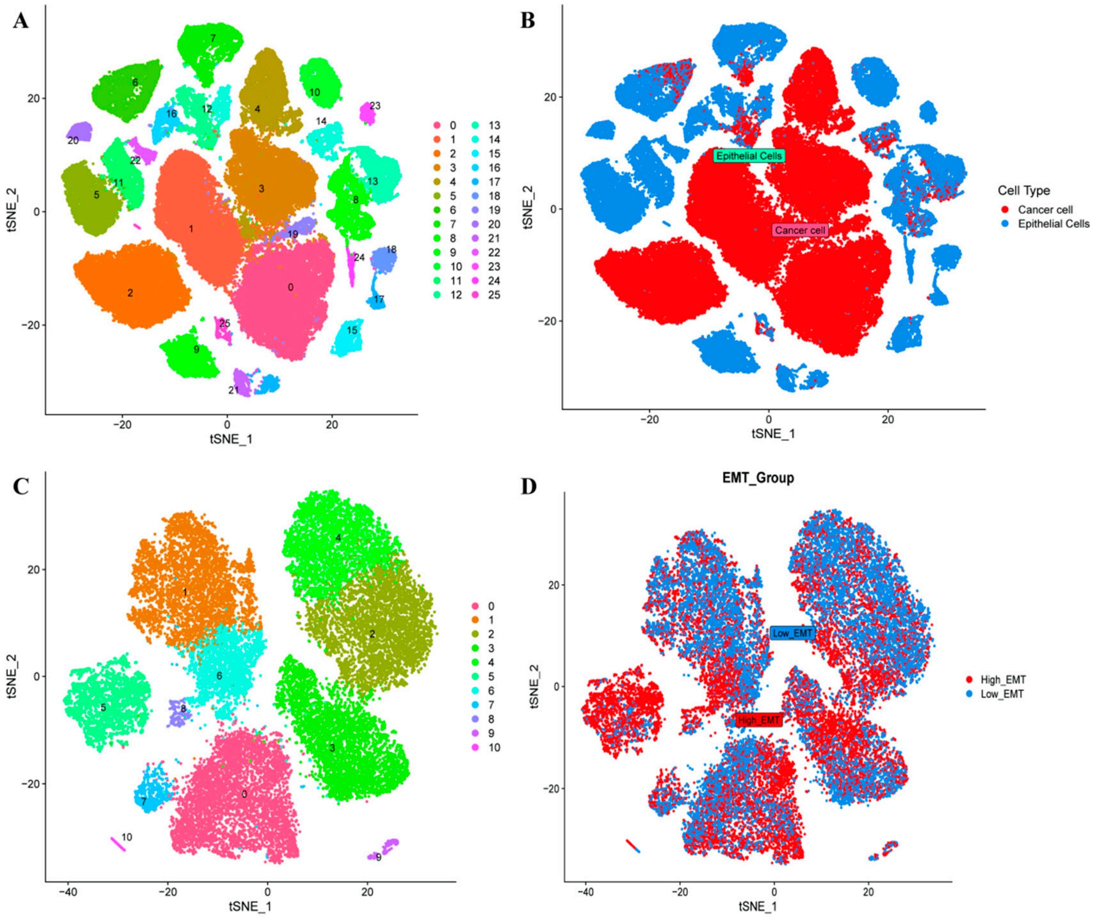

Supplementary Figure S2. Workflow for selection and grouping of EMT-related malignant epithelial cells. (**A**) tSNE plot of epithelial cells after extraction, showing the distribution of epithelial cell subpopulations. (**B**) Annotation of CopyKAT inference results, classifying epithelial cells into malignant and benign cells. (**C**) tSNE plot of malignant epithelial cells, showing their cell population distribution. (**D**) Annotation of malignant cell groups based on AUCell EMT scores (EMT-high vs. EMT-low).

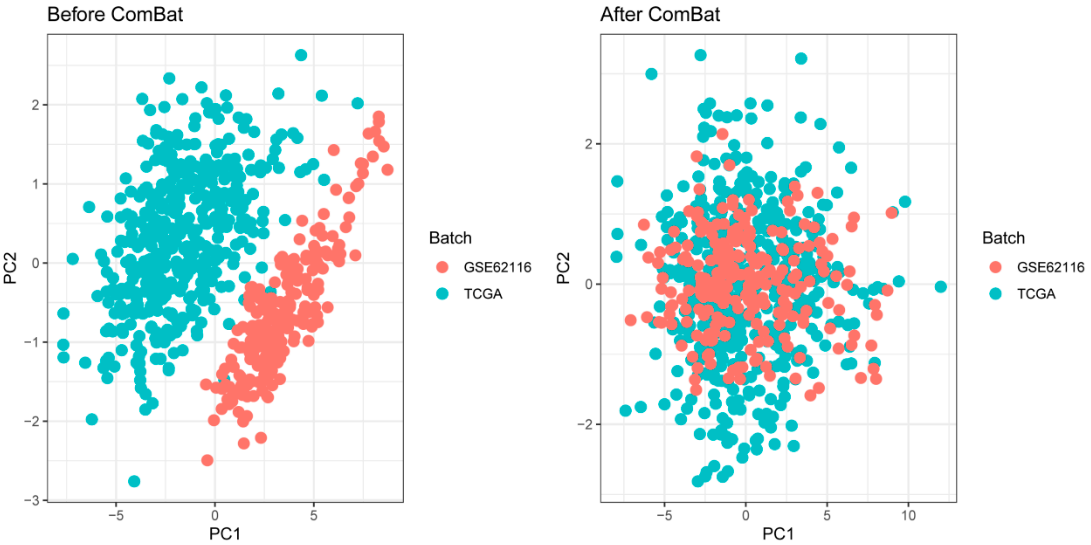

Supplementary Figure S3. PCA before and after batch effect correction using ComBat in TCGA-PRAD and GSE62116 cohorts.

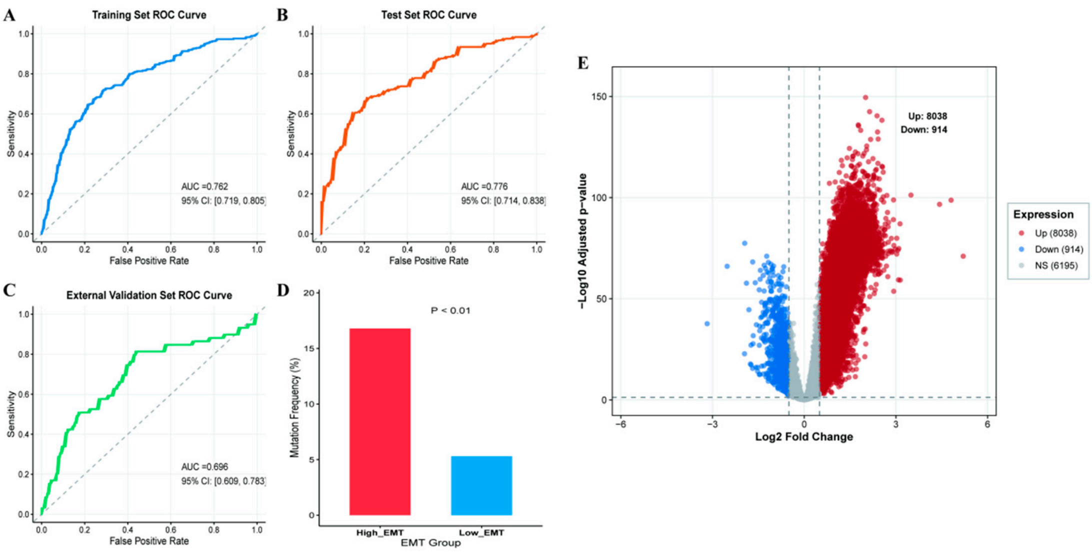

Supplementary Figure S4. EMT-related risk model and functional analysis results. (**A**) Time-dependent ROC curves for patients in the model training cohort. (**B**) Time-dependent ROC curves for patients in the model testing cohort. (**C**) Time-dependent ROC curves for patients in the external validation cohort. (**D**) Bar plot comparing TP53 mutation frequency between different EMT infiltration score groups. (**E**) Volcano plot of DEGs between different EMT infiltration score groups, showing significantly up- or down-regulated genes.

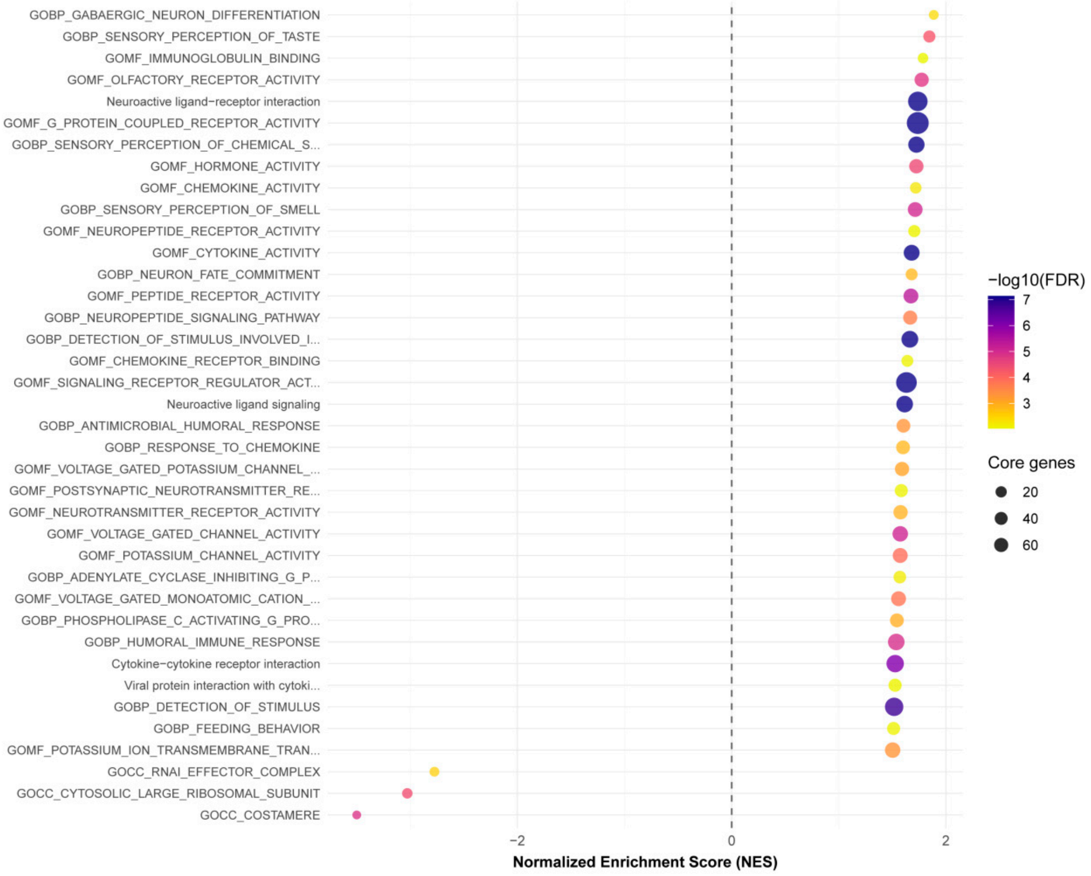

Supplementary Figure S5. Top 20 pathways enriched by GSEA. Bubble plot of the top 20 pathways from GSEA, showing functional pathways significantly enriched in high- and low-EMT score groups.

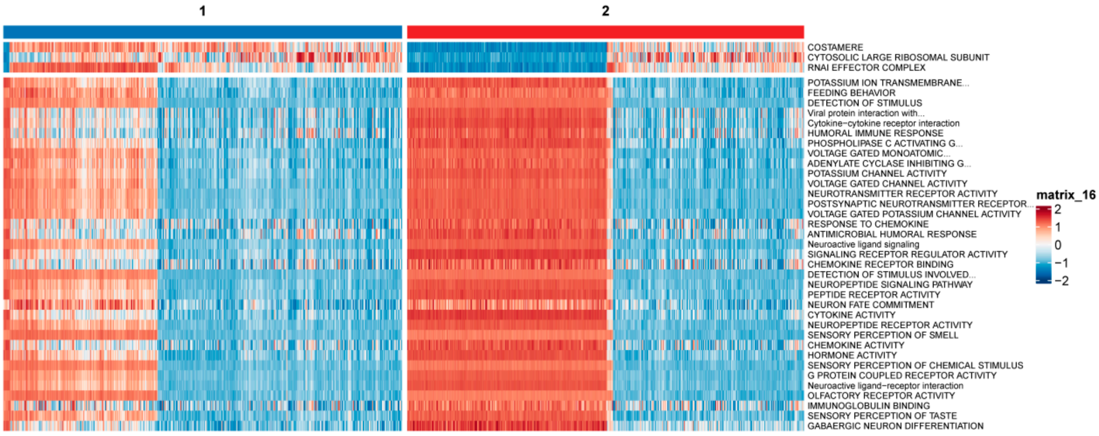

Supplementary Figure S6. GSVA pathway enrichment heatmap. Heatmap of sample pathway enrichment from GSVA analysis, showing differences in activity levels of multiple functional pathways across samples.

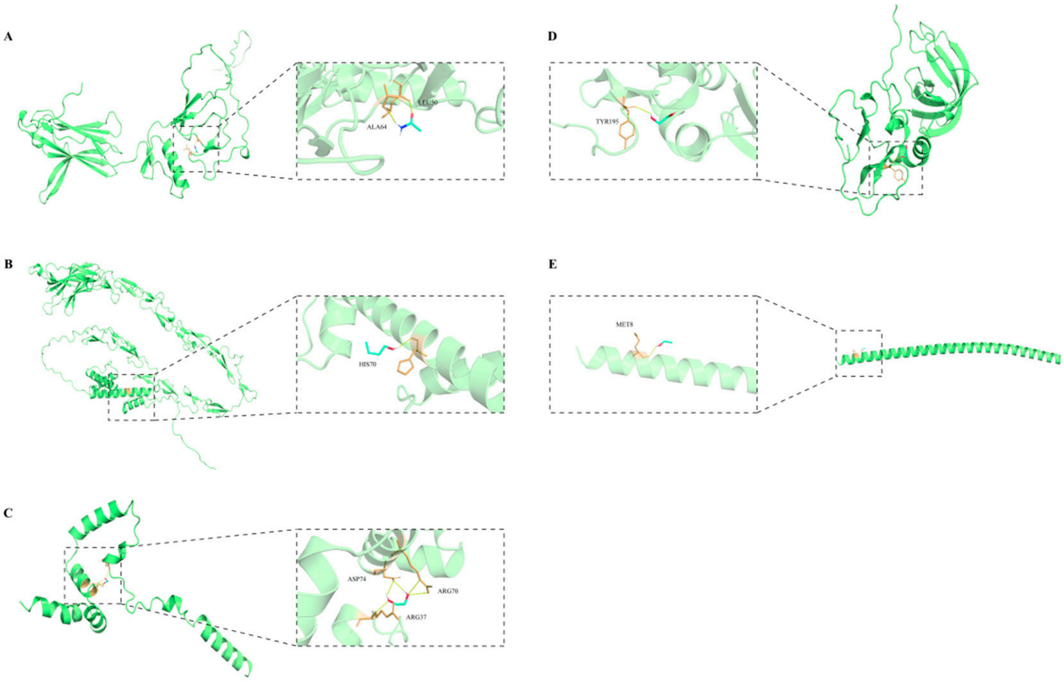

Supplementary Figure S7. Supplementary Figure 7. Molecular docking results between core EMT-related proteins and negative control compounds. (**A**) Molecular docking mode of IGFBP7 with Acetamide (binding affinity = −2.6 kcal/mol). (**B**) Molecular docking mode of FBLN1 with 1-Butanol (binding affinity = −3.1 kcal/mol). (**C**) Molecular docking mode of MGP with Ethylene Glycol (binding affinity = −2.7 kcal/mol). (**D**) Molecular docking mode of TIMP3 with Ethylene Glycol (binding affinity = −3.2 kcal/mol). (**E**) Molecular docking mode of TPM2 with Ethanol (binding affinity = −1.8 kcal/mol).
